# Pulmonary thromboembolism and obesity in forensic pathologic case work

**DOI:** 10.1007/s12024-023-00602-9

**Published:** 2023-03-21

**Authors:** Michael Klintschar, Kirsten Wöllner, Lars Hagemeier, Theresa A. Engelmann, Jan Mahlmann, Alessia Lunow, Roman Wolff-Maras

**Affiliations:** Institute of Forensic Medicine, Hanover Medical School, Department of Legal Medicine, Carl-Neuberg-Straße 1, 30625 Hannover, Germany

**Keywords:** Autopsy, Pulmonary embolism, Body mass index, Association, Subcutaneous adipose thickness

## Abstract

328 autopsy cases of fatal pulmonary thromboembolism (PE) were compared to 984 age- and sex-matched controls to evaluate the association between obesity and PE in a forensic context. Both PE and control cases had a mean age of 67,8 years (male 62,9 years, females 71,7 years). The percentage of morbidly obese persons with a body mass index (BMI) of above 40 or abdominal subcutaneous adipose tissue of above 4 cm was higher in the PE group (8,39% vs. 4,67% and 29.45% vs. 23.40%, respectively). On the other side, that of very slim persons (BMI below 18.5 or adipose tissue below 3 cm) was significantly smaller (4,27% vs. 7,52% and 47.55% vs. 56,60%). We thus found a strong association between being overweight and death from PE, while slim persons seem to be at an advantage. As the group of underweight persons includes those suffering from chronic diseases with reduced mobility or hypercoagulability (e.g. tumor kachexia or sarkopenia due to immobilisation), this finding is to some extent unexpected.

Pulmonary embolism (PE) is an (in many instances lethal) complication of venous thromboembolism (VTE). PE is frequently observed when performing forensic autopsies and is thus mentioned in standard textbooks as well as objective of research in forensic medicine [1–4]. Also genetic factors contributing to the risk of PE have been studied in the forensic context [5–7].

Besides a genetic component, the many risk factors include cancer, surgery, trauma, immobilization, pregnancy, oral contraceptive use, old age etc. [8].

One further factor discussed to be associated with PE is obesity [9, 10], a condition of increasing frequency throughout the world [11, 12].

As a simple screening tool for differing between normal and abnormal weight, in many instances the Body Mass Index, or BMI, is used. It is defined as a person’s weight in kilograms divided by the square of height in meters. According to the World Health Organization, a BMI of 18.5 to < 25 is considered normal. A BMI of 25.0 to < 30 is considered overweight, while a BMI below 18.5 is underweight. Persons with a BMI of above 30 are considered to be obese and those above 40 morbidly obese.

An association between PE and obesity was initially described in 1927 [13] and was confirmed in large clinical studies such as the Nurses´ Health study [14] (in that women aged 30–55 years with a BMI of above 29 had a relative risk of 2.9 to develop PE compared to women of normal weight). Nevertheless, the association between obesity and PE might be less straightforward than initially assumed, at least when considering the fatal cases: A paradoxical beneficial effect of severe overweight was reported recently with a lower mortality from PE in obese persons compared to persons with normal or low weight [15]. In this study, obese persons with venous thromboembolism showed less than 50% the mortality rate of persons with normal BMI. This mortality paradox was confirmed by several subsequent studies [16, 17].

It is well known that PE is a difficult clinical diagnosis [18] and in many instances deaths from PE are diagnosed only upon autopsy: Among those patients who die of PE, 94% do so before diagnosis [19]. The well known decline in the clinical autopsy rate has thus resulted in an increased number of PE cases remaining undiagnosed [20]. We conclude from this that studies that predominantly rely on clinical data, but not on autopsy data, might be biased.

Up to now, there are only very few studies on the association of obesity and deaths from PE, in which a complete autopsy was performed in all patients: A study on pathologic autopsies of persons deceased in hospitals in Malmö between 1970 and 1982 found an increased risk of death from PE in persons with a BMI of above 22.4 (OR 1.24), in persons with an abdominal subcutaneous fat tissue thickness of more than 20 mm (OR 1.28) and a thoracic subcutaneous fat tissue thickness of more than 9 mm (OR 1.35).

In the forensic context, there is only one pertinent study: Rosenfeld et al. compared 160 deaths from PE and 160 other forensic autopsy cases from Australia and found an average BMI of 30,88 in PE deaths compared to 25,33 in other autopsies [21]. They report very strong correlations between BMI and death from PE.

Nevertheless 160 cases is a rather small group and a larger study might lead to a better estimation of the role of obesity in PE mortality. Therefore, to further deepen the insights into the relation of body weight and risk to die from PE in the forensic case work, we analyzed a considerably larger autopsy population from Germany.

## Patients and methods

The Institute of Legal Medicine in Hannover and Oldenburg performed 7150 autopsies in the years January 2012- April 2019. These autopsy protocols were retrospectively studied and altogether 328 cases identified, in which death was unambiguously caused by PE. The cases included 143 male and 185 female persons. Body height and body weight were measured before autopsy. During autopsy the maximal thickness of the abdominal subcutaneous adipose tissue (SC) was measured. Deceased persons with significant putrefaction were excluded from the study as well as persons younger than 18 years. The PE cases had a mean age of 67,8 years (men 62,9 years, females 71,7 years). The autopsy protocols included a forensic/criminalistic/medical case history which included the major preexisting diseases and circumstances of death, but usually not the medication. From these informations 5 major risk factors were identified: physical inactivity, recent trauma, recent operations, tumor disease and other causes. The frequency of these risk factors is given in Table 1

**Table 1:** The frequency of common risk factors besides obesity in the 328 PE cases (143 males and 185 females)

	**immobilization**	**surgery**	**trauma**	**tumor**	**others**	**none**	
male	13,3%	34,3%	18,2%	14,0%	3,5%	36,4%	
female	10,8%	29,2%	28,1%	11,4%	2,7%	38,9%	
total	11,9%	31,4%	23,8%	12,5%	3,0%	37,8%	

As control sample for each PE case three cases that had died from other causes were randomly selected. These samples had the same age (in years) and sex as the causes. The control group thus contained 984 deceased persons of the same age and sex distribution as the cases.

## Statistics

The autopsy data were stratified according to sex. Mean BMI and thickness of the subcutaneous adipose tissue (SC) were calculated and compared to the autopsy control group using a chi square test (https://www.medcalc.org/calc/comparison_of_means.php).

Relative risks for different weight groups were calculated using the online relative risk calculator (https://www.medcalc.org/calc/relative_risk.php). Both tools are made available online by MedCalc.

## Results

The PE group had an average BMI of 28.75 and a mean adipose tissue thickness of 3,77 cm. The Non-PE group had a significantly smaller BMI of 26.72 and 3.34 cm adipose tissue (Table 2). Interestingly, the BMI differences were more prominent in men than in women (29,25 vs. 26,78; 28,32 vs. 26,67) while they were significant in both groups. The SC fat thickness differences were, however, comparable for men and women (3,69 vs. 3,26; 3,82 vs. 3,40). Moreover, the p-values for the comparisons between PE group and controls for subcutaneous fat tissue were smaller than those for BMI, indicating that BMI might be closer correlated to PE than the SC fat tissue.

**Table 2:**
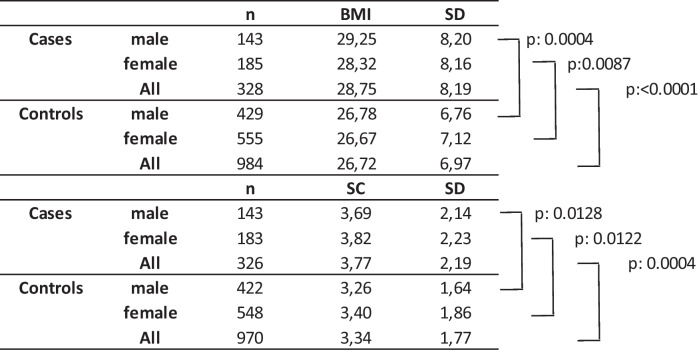
Body mass index and subcutaneous fat tissue in deaths from PE (cases) and other causes of death (controls). *BMI* mean body mass index, *SC* thickness of the subcutaneous fat tissue (cm) *SD* standard deviation

As given in Table 3, the percentage of morbidly obese persons with a BMI above 40 or a SC adipose tissue above 4 was larger in the PE group (in the combined group 8,39% vs. 4,67% and 29.45% vs. 23.40%, respectively), while that of very slim persons (BMI below 18.5 or sc adipose tissue below 3 cm) was smaller (4,27% vs. 7,52% and 47.55% vs. 56,60%). Using these data, relative risks were calculated (Table 4), that were significantly increased for persons (males and females) with a BMI above 40. A deleterious effect of a BMI above 30 or 25 could not be demonstrated. For the SC thickness > 4 cm only in the combined group a significantly increased risk was calculated. Interestingly, we could demonstrate a significant protective influence of being underweight (BMI below 18.5 or sc adipose tissue < 3 cm) in the combined male and female group.

**Table 3:** The distribution of BMI and subcutaneous tissue thickness groups in cases and controls

**BMI**		**< 18.5**	**18,5–25**	**25–30**	**30–40**	**> 40**
**Cases**	**male**	2,10%	26,57%	39,16%	23,78%	8,39%
	**female**	5,95%	31,89%	28,11%	22,16%	11,89%
	**All**	4,27%	29,57%	32,93%	22,87%	10,37%
**Controls**	**male**	4,90%	38,46%	31,00%	22,38%	3,26%
	**female**	9,55%	35,86%	28,29%	20,54%	5,77%
	**All**	7,52%	36,99%	29,47%	21,34%	4,67%

**SC**		**< = 3 cm**	**3—4 cm**	**> 4 cm**		
**Cases**	**male**	49,65%	23,08%	27,27%		
	**female**	45,90%	22,95%	31,15%		
	**All**	47,55%	23,01%	29,45%		
**Controls**	**male**	57,11%	22,51%	20,38%		
	**female**	56,20%	18,07%	25,73%		
	**All**	56,60%	20,00%	23,40%		

*BMI* mean body mass index, *SC* thickness of the subcutaneous fat tissue (cm)

**Table 4:** Relative risks calculated from the data in Table 3

**BMI**	**< 18.5**	**18.5–25**	**25–30**	**30–40**	**> 40**
male	n.s	n.s	n.s	n.s	1.2177 to 5.4300 p:0.0133
female	n.s	n.s	n.s	n.s	1.2302 to 3.4578 p:0.006
All	0.325 to 0.991 (p:0.0464)	n.s	n.s	n.s	1.4492 to 3.3927 p:0.0002

**SC**	**< = 3 cm**	**3—4 cm**	**> 4 cm**		
male	n.s	n.s	n.s		
female	n.s	n.s	n.s		
All	0.7401–0.9535; p:0.0070	n.s	1.0272–1.5415; p:0.0265		

*n.s.* not significant, *BMI* mean body mass index, *SC* thickness of the subcutaneous fat tissue (cm)

## Discussion

The frequency of PE in autopsy studies, both clinical and forensic, is reported to vary between 3 and 20% [22]. We found a fatal PE in 328 of the 7150 autopsies performed at our institute during more than 7 years (4,5%) and thus PE was relatively rare in our casework. When considering common well known risk factors (besides obesity) like immobilization, trauma, surgery or tumor disease, such factors were reported for more than 2/3 of the cases (Table 1).

For clinical autopsies, the only study was published more than 20 years ago [23]. Also in the forensic context up to now only one such study was published: Rosenfeld et al. compared 160 deaths from PE and 160 other forensic autopsy cases from Australia and found an average BMI of 30,88 in PE deaths compared to 25,33 in other autopsies [21].

Our study with 328 cases and 984 cases is thus the largest autopsy based investigation on the association of pulmonary embolism and adiposity in the forensic case work that has been published do date.

We could confirm the results of the study by Rosenfeld et al. insofar as we also found a strong association between overweight and death from PE. However, our results were less unambiguous: The BMI of our PE cases was lower than that of Rosenfeld et al. (28.75 vs. 30.88), whereas our controls were slightly more adipose (26,72 vs. 25.33). Moreover, in the Australian study a correlation between BMI and death from PE was reported for persons with a BMI of above 30, while in our study the effect was significant with a BMI of above 40, but not in persons with relatively moderate obesity.

On the other hand, we could demonstrate that very slim persons (BMI < 18.5 and SC adipose tissue < 3 cm) are at a decreased risk to succumb from PE. As underweight is in many instances the consequence of chronic diseases with reduced mobility or hypercoagulability (e.g. tumor kachexia or sarkopenia due to immobilisation), this finding is to some extent unexpected: Barba et al. e.g. report a higher mortality in underweight persons [15], although Rahmani reports a protective influence of being underweight against VTE [24]. Although we were able to identify several risk factors in our study group, the number of underweight persons is small (< 5%) and thus no detailed analysis in that respect was possible.

Several reasons could be responsible for the differences between the study by Rosenfeld et al. [21] and our study: On one hand the proportion of obese persons is higher in Australia: According to Wikipedia (https://en.wikipedia.org/wiki/List_of_countries_by_body_mass_index), the average BMI is 27.2 in Australia, but 26.3 in Germany. On the other hand, the composition of the cases on which forensic autopsies are performed varies between different centers: Our German group has a high proportion of medical malpractice cases and sudden unexpected deaths, whereas accidents or homicides are relatively rare. The proportion of obese persons in these groups might vary widely. We do not know the composition of the Australian study, but argue that such a difference might explain our weaker association. And finally, our study is significantly larger, a fact that should (positively) influence the informative value.

The mechanism by which obese persons are predisposed to PE is believed to be multifactorial [25]: On one hand, a high BMI is often associated with lack of activity, restricting the emptying of the veins of the leg. Thus, obesity might be an indirect risk factor, via the degree of activity.

On the other hand, visceral obesity causes an increased intra-abdominal pressure, leading to increased pressure in the femoral veins, which favors stasis in the deep veins of the legs [26].

Even more so, adiposity is known to have direct influence on the coagulability of blood. E.g. the thrombozytes are enlarged, which promotes clotting. The adipocytes release adipokines which are bioactive peptides that influence the function of several organ systems. These factors include tumor necrosis factor alpha and interleukin 6 [27], cytokines that mediate an inflammatory reaction, endothelial damage and finally introduce a prothrombotic state [28].

One inherent problem with the BMI is that it cannot discriminate between a person with a high proportion of fatty tissue and a very muscular person. Moreover, it does not account for the distribution of fat tissue over the body. In clinical studies the BMI thus is only one of several anthropomorphic markers use to predict individual disease risks, others include total fat, waist-to –hip ratio or waist circumference [29]. We decided to include the thickness of the abdominal subcutaneous fat tissue into the present study, as done so in a former study on clinical autopsies [23]. Nevertheless, we found this parameter to be an inferior predictor of the relative risk to succumb from PE. We therefore conclude that the BMI (that is very easy to determine) might be a suitable anthropomorphic marker for studies on the correlation between obesity and morbid complications.

In conclusion, we report a significantly higher BMI in persons that had died from PE compared to other deaths in the forensic case work. We did not observe the beneficial effect of increased BMI reported in large clinical studies (without performing autopsies). The subcutaneous fat tissue, a marker that might be more closely related to the waist-to-hip ratio, was also associated with the risk to die from PE, but the correlation was weaker than that of the BMI. We found a lower risk for persons with a BMI below 18.5. This finding should be investigated further in a larger group.

## Key points

1. Pulmonary thromboembolism (PE) is commonly observed in forensic autopsies (3–20%).
2. Morbid obesity (BMI > 40) is a significant risk factor for death from PE.
3. Lack of mobilization, stasis in the femoral veins and higher coagulability via adipokines might all contribute to this risk.
4. At least in the forensic context, the risk of very slim persons (BMI < 18.5) is lower than that of persons with normal weight.
5. Subcutaneous fatty tissue depth is a poorer estimator of the risk to succumb from PE than BMI.

## Data Availability

The original data are available from the authors upon request.
